# Diabetogenic glucose and insulin concentrations modulate transcriptom and protein levels involved in tumour cell migration, adhesion and proliferation

**DOI:** 10.1038/sj.bjc.6606050

**Published:** 2010-12-21

**Authors:** K Masur, C Vetter, A Hinz, N Tomas, H Henrich, B Niggemann, K S Zänker

**Affiliations:** 1 Institute of Immunology and Experimental Oncology, University Witten/Herdecke, Stockumer Str. 10, Witten 58448, Germany

**Keywords:** hyperglycaemia, hyperinsulinemia, Warburg effect, proliferation, adhesion, cell migration

## Abstract

**Background:**

During the last decade, epidemiological studies uncovered the tremendous impact of metabolic syndrome/diabetes mellitus type 2 (DM T2) as risk factors of the progression of cancer. Therefore, we studied the impact of diabetogenic glucose and insulin concentrations on the activities of tumour cells, because little is known about how high glucose and insulin levels are influencing gene activities causing changes in the signal cascade activities with respect to kinases involved in the proliferation and migration of cancer cells.

**Methods:**

To address this question we analysed the activity of more than 400 gene signatures related to (i) cell cycle, (ii) cell movement as well as (iii) signal transduction. We examined transcriptoms of kinases (PKC*α*, PI3K), cadherins (E-, N- VE-), integrins and cyclins by comparing physiological (5.5 mM) *vs* diabetogenic (11 mM) glucose concentrations (without and with insulin).

**Results:**

Proliferation assays revealed that high levels of glucose (11 mM) and insulin (100 ng ml^−1^) did promote the proliferation of the tumour cell lines HT29, SW480, MCF-7, MDA MB468, PC3 and T24. Using a 3D-migration assay, we have shown that high glucose concentrations caused increased motility rates of the tumour cells. The increase in migratory activity at high glucose and insulin concentrations was mediated by an activation of PI3K, PKC*α* and MLCK, as figured out by the pharmacological inhibitors wortmannin, Go6976 and ML-7.

**Conclusion:**

We present molecular and functional data, which could help to understand how hyperglycaemia and hyperinsulinemia might trigger tumour cell proliferation and motility in patients, too.

Over the past few decades the number of people with excess body weight has been increasing in both industrialised and developing countries. About 50% of men and 35% of women in Europe are currently estimated to be overweight or obese. Bianchini *et al* (2002) showed that excess body weight is directly associated with risk of cancer at several organ sites, including colon and breast.

Latest epidemiological studies indicated a connection between obesity/diabetes mellitus type II (DM T2) and increased appearance of new tumour cases. Adiposity is leading to increased serum glucose, insulin and triglycerides levels, which are risk factors for cardiovascular disease and DM T2. Retrospective (Krone and Ely, 2005) and prospective (Stattin *et al*, 2007) studies indicating a direct influence of high glucose and triglycerides on the progression of cancer. The outcome, if these epidemiological data is that metabolic alterations leading to obesity, insulin resistance and adiposity are not only risk factors to get DM T2 but also risk factors for various kinds of cancer (Giovannucci, 2001; Keku *et al*, 2005). There is growing evidence that the metabolic syndrome is a common nominator for hypertension, DM T2 and some types of cancer (Brauer *et al*, 2002; Vona-Davis *et al*, 2007).

Besides an increasing number of epidemiological investigations, there are less experimental studies investigating the molecular background between metabolic alterations and the progression of cancer (Yin *et al*, 2005; Langbein *et al*, 2006). Nevertheless, since more than two decades it is known that glucose is the driving force for the growth of tumour cells (Beckner *et al*, 1990) and that increased insulin levels promote metastasis (Stracke *et al*, 1988). However, little is known about the effects of increased glucose and insulin concentrations on changes at the transcriptom level or on the modulation of signalling cascades involved in cell motility or adhesion. Recent *in vitro* studies suggest that an important mechanism of enhanced glucose metabolism in carcinoma cells involves the overexpression of transmembrane glucose transporters (Rudlowski *et al*, 2004; Hauptmann *et al*, 2005). Changes in glucose metabolism have also been found in many tumours, causing an increased production of lactate (Walenta *et al*, 2000; Walenta and Mueller-Klieser, 2004). Elevated lactate within cancer cells indicates a switch in glucose metabolism from aerobe to an anaerobe utilisation of glucose, first described by Warburg (1956). Modern molecular biology leads to a renaissance of the Warburg effect (Kim and Dang, 2006; Kondoh, 2008). A permanent increase of anaerobe glucose utilisation in primary tumours characterizes more aggressive tumour cells (Walenta *et al*, 2001). The reduced mitochondrial respiration and an increased conversion of glucose into lactate combined with an enhanced secretion of lactate is associated with an acidification of the tumour and its environment (Pelicano *et al*, 2006). This turns into an advantage for tumour cells with a resistance to acidosis, realized by an increased H^+^ transporter activity (e.g. N^+^/H^+^ exchanger) (Gatenby *et al*, 2007). However, in non-malignant tissues, an acidic microenvironment is ordinarily toxic to mammalian cells typically resulting in apoptosis through activation of caspases (Thews *et al*, 2007). The persistence of acidosis is leading to the destruction of the extra cellular matrix surrounding the tumour – possibly further supported by the acid-mediated activation of matrix metalloproteinases (Chaussain-Miller *et al*, 2006), and thereby promoting migration of tumour cells, a prerequisite of metastasis formation (Langbein *et al*, 2006).

Here, we report about changes in proliferation, locomotion and gene signatures of tumour cells cultured under diabetogenic conditions when compared with physiological glucose concentrations. Moreover, the addition of insulin to hyperglycemic-grown tumour cells further increased proliferation, as well as cell migration, by modifying the expression of molecules involved in cell cycle regulation, signal transduction, cell–cell adhesion and cellular locomotion.

## Materials and methods

### Cell culture and treatment

All cell lines were cultured at three different conditions in parallel: RPMI1640 either containing 5.5 mM or 11 mM glucose, or RPMI with 11 mM glucose plus 100 ng ml^−1^ insulin. Each medium was supplemented with 10% FBS, 1% penicillin/streptomycin, and cells were kept at 37°C in a humidified atmosphere (5% CO_2_). After an adaptation of at least two passages at the appropriate media, cells were used for experiments.

### Three-dimensional cell migration assay

Cultured cells were harvested using a trypsin/EDTA solution and 100.000 cells were mixed with 150 *μ*l buffered liquid collagen (pH 7.4, 1.63 mg ml^−1^ collagen type I; Collagen Corporation, Fremont, CA, USA) containing minimal essential medium (Sigma, Deisenhofen, Germany) and the investigated substances: insulin, wortmannin, ML-7 (Sigma) or Go6976 (Calbiochem, Bad Soden, Germany), as described previously (Friedl *et al*, 1993). After polymerization the chambers were sealed, and locomotion was recorded by time-lapse video-microscopy 12 h at 37°C. For analysis of the migratory activity, 30 cells per sample were randomly selected, and projections of paths were digitised as x/y-coordinates in 15-min intervals. The average migratory activity was calculated for each 15-min interval (100% migratory activity is reached, when all 30 cells migrate all over the time).

### Proliferation assay

On day 0, 100.000 cells were seeded into 24-well plates and incubated at physiological conditions (5.5 mM), or at diabetogenic glucose concentrations (11 mM) alone or in combination with 100 ng ml^−1^ insulin, respectively. Each day, two wells per sample were trypsinized and counted to establish cell growth curves. To determine proliferation rates, exponential phases of each cell growth curve were used for calculations. A MTT proliferation assay (CellTiter96 Aqueous One Solution Cell Proliferation Assay/Promega, Madison, WI, USA) was performed according to the manual, to confirm data received from cell growth curves.

### Gene activity/DNA microarray

Tumour cells were cultured at 5.5 mM glucose, 11 mM glucose or 11 mM plus 100 ng ml^−1^ insulin. Customised gene chips for DNA microarray analysis were purchased from Ocimum Biosolutions Ltd, Hyderabad, India. The mRNA (5 *μ*g) was prepared using a Macherey & Nagel Nucleo Spin RNA 2 Kit (Macherey-Nagel Gmbh & Co KG, Düren, Germany), with a subsequent cDNA transcription. Each Cy-3-labelled sample was combined with the Cy-5-labelled control and hybridised onto customised Ocimum 40k arrays for 16 h at 65°C. Hybridised arrays were scanned and quantified using the Agilent Feature Extraction Software 7.1. (Agilent Technologies Deutschland GmbH, Böblingen, Germany). Data were excluded for genes that did not have an expression signal above 200 in either channel. Significance was determined by an at least 2.5-fold change in expression. Each experiment was repeated three times.

### Immunoblotting

The expression levels of E-cadherin, MLCK and PKC*α* were analysed by immunoblotting as described previously (Masur *et al*, 2001). Cells (2.5 × E5 per sample) were lysed in Laemmli sample buffer and incubated for 10 min at 95°C. Proteins were separated using PAGE according to Laemmli (Laemmli, 1970) and were transferred to an Immobilion-P membrane (Millipore, Bedford, MA, USA), followed by blocking the membranes with 10% milk powder (1 h at RT). After incubating the membrane (over night at 4°C) with the primary monoclonal antibodies against E-cadherin (clone 67A4), purchased from Coulter-Immunotech (Krefeld, Germany), the goat polyclonal antibody against MLCK (MYLK (P-19): sc-22224; Santa Cruz Biotechnology Inc., Heidelberg, Germany) or PKC*α* (Transduction Laboratories, Lexington, KY, USA), membrane was washed vigorously with PBS-Tween. Subsequently, the membrane was incubated with either anti-mouse or anti-goat peroxidase-linked antibody (1 h at RT), followed by the chemiluminescence substrate (1 min at RT) (Roche Diagnostics, New Castle, IN, USA). Bands were visualised using the BM chemiluminescence blotting substrate (Roche Diagnostics GmbH, Mannheim, Germany) and detected by Aequoria Macroscopic Imaging (Hamamatsu Photonics, Herrsching am Ammersee, Germany).

### Flow Cytometry

The expression of integrins and cadherins was analysed by flow cytometry (antibodies see above). The IgG1 isotype and secondary FITC-conjugated goat anti mouse or anti-rabbit antibody were obtained from New England Biolabs, Frankfurt, Germany. The mean fluorescence intensity of the bound antibody at 11 mM glucose (±insulin) was compared with the binding of the same antibody at 5.5 mM.

### PKC activity assay

The tumour cells (2.5 × E5 per sample) were cultured either in 5.5 mM, 11 mM glucose or 11 mM glucose plus 100 ng ml^−1^ insulin, and afterwards treated according to the manufacturer's protocol (StressXpress PKC Kinase Activity Assay Kit (EKS-420A), Biomol GmbH, Hamburg, Germany). In brief, cells were lysed, centrifuged and supernatants were added to PKC-substrate-coated wells. After incubation with ATP, a phospho-specific HRP-conjugated antibody was added, and finally TMB was used for colorimetric determination of PKC activity (Shirai and Saito, 2002). Absorbance of the positive control was used to calculate the amount of active PKC.

### Data analysis

Statistical comparison between specified values was made by ANOVA using a Dunnett's multiple comparisons test or the student's *t*-test. Significances are indicated by the stars: ^*^P<0.05, ^**^P<0.01, ^***^P<0.001; NS, not significant (*P*>0.05).

## Results

To analyse the proliferation capacity, we cultured tumour cells lines of different organs (HT29 and SW480 colon carcinoma; MCF-7 and MDA MB468 breast cancer; PC3 prostate cancer; T24 bladder carcinoma) at physiological and diabetogenic glucose concentrations without and with the addition of 100 ng ml^−1^ insulin (see Table 1A). Almost all tumour cell lines revealed higher proliferation rates when cultured under hyperglycemic conditions, and all cell lines showed increased proliferation after the addition of 100 ng ml^−1^ insulin. Although all cell lines displayed decreased proliferation at low glucose concentrations of 2.75 mM, there were differences in the proliferation rate at physiological (5.5 mM) and diabetogenic glucose concentrations (11 mM and 22 mM). HT29 and MCF-7 cells steadily increased the number of cells when glucose concentration was elevated, whereas SW480 and T24 showed increased proliferation until a glucose concentration of 11 mM, and at 22 mM glucose proliferation dropped below the 11 mM level. However, PC3 cells showed no significant differences in proliferation in relation to glucose concentration. Furthermore, all tumour cells grown in medium containing additional 100 ng ml^−1^ of insulin (with 5.5 mM or 11 mM glucose) showed a pronounced increase of proliferation – displaying on average 20–40 percent higher proliferation rates. Cell growth curves revealed doubling times of 2.4 days for MDA MB468 cells when cultured at normoglycemic conditions, 1.9 days at 11 mM glucose and 1.4 days when cultured in 11 mM glucose plus 100 ng ml^−1^ insulin (data not shown). Comparable results were found in SW480 colon carcinoma cells with 1.9 days at 5.5 mM glucose *vs* 1.5 days at 11 mM glucose and 1.4 days at 11 mM plus 100 ng ml^−1^ insulin. Furthermore, also migratory activity was increased by high levels of glucose and insulin. Table 1 displays the elevated migratory activity (1B) and the increased distance migrated (1C) of the investigated cancer cells. Both effects activity and distance were strongly dependent on glucose concentrations. All cell lines displayed an increased duration of migratory activity, resulting in an enhanced distance of migration. Especially the shortening of the pauses between periods of cell movement led to glucose- and insulin-induced increases of migratory activity and therefore to enhanced distances.

In MDA MB468 cells, we observed almost a doubling of the distance migrated (18 *μ*m *vs* 33 *μ*m) comparing cells cultured in 5.5 mM glucose or in 11 mM glucose. At both glucose concentrations, addition of 100 ng ml^−1^ insulin further increased the distance migrated (36 *μ*m for 5.5 mM plus insulin and 58 *μ*M for 11 mM plus insulin). Therefore, an increase of glucose- and insulin concentration caused a three-fold elevated distance migration of MDA MB468 cells. Both, glucose-mediated and insulin-induced stimulation of locomotory activity could be reduced with the PKC*α*-specific inhibitor Go6976 (6 nM) to the level of naïve cells.

DNA-microarray analysis revealed significant changes (>2.5-fold) in gene activity of numerous genes involved in proliferation, adhesion and migration (Table 2). As shown in Table 2, the most pronounced increase in gene activity was found for cyclin A1: 14-fold increase at 5.5 mM *vs* 11 mM glucose. We also found increased numbers on transcriptoms of genes involved in signal transduction and locomotion (PKC alpha and delta, integrins beta1 and alpha2 and several myosins). Interestingly, genes for cell adhesion were differentially regulated. We found a four-fold downregulation of E-cadherin on the one hand, but in parallel a three-fold to five-fold upregulation of cadherins of the vascular and neuronal subclass (Table 2) at diabetogenic glucose and insulin concentrations.

**Figure 1:**
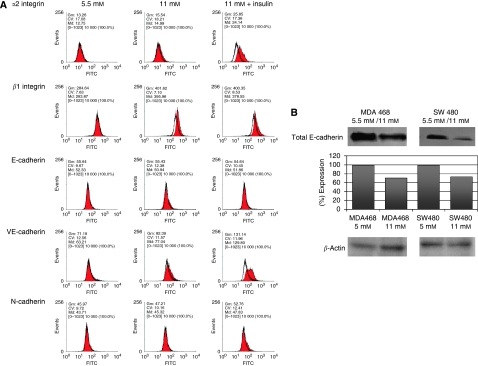
(**A**) Cell surface expression levels of *α*2 and *β*1 integrin, E-, N- and VE-cadherins on SW480 colon carcinoma cells. The antibody-bound FITC-fluorescence of specific antibodies at 11 mM glucose or 11 mM glucose plus insulin (filled grey) was directly compared with 5.5 mM control cells (grey line) as well as to the isotype control (black line). Significant increases (more than 15%) have been found for *α*2, *β*1integrins and for VE-cadherin. For N-cadherin only a marginal induction was detectable, whereas E-cadherin showed no significant changes. The geometric means (Gm) indicated the fluorescence intensity of the FITC-conjugated antibodies. (**B**) Immunoblot analysis of E-cadherin expression of breast cancer (MDA MB468) and colon carcinoma (SW480) cell lines. Antibodies against the cell–cell contact molecule E-cadherin were used, and specifically bound antibodies were detected with the use of a secondary HRP-linked mouse anti-human antibody. The expression levels of all blots were evaluated using the Wasabi software version 1.4 (Hamamatsu Photonics, Herrsching am Ammersee, Germany).

To confirm these results at the protein level, we used flow cytometry and western blot analysis, to determine the integrin and cadherin protein expression levels (Figure 1). Figure 1A depicts altered expression levels of alpha-2 integrin, beta-1 integrin, E-cadherin, VE-cadherin and N-cadherin at physiological (5.5 mM, left column) glucose concentrations, high glucose concentrations (11 mM, middle column) and diabetogenic conditions (11 mM plus insulin, right column).

Using flow cytometry, we found an upregulation of alpha-2/beta-1 integrins, the collagen receptor, when cells were cultured at high glucose. Alpha-2/beta-1 integrin expression level was elevated at diabetogenic condition (alpha-2 subunit: 1.2-fold and beta-1 subunit: 1.4-fold). When cells were cultured in high glucose and high insulin concentration, the integrin expression was significantly increased (alpha-2 integrin subunit: two-fold and beta-1 integrin subunit: 1.4-fold). This is in accordance with our results found for the increased number of transcriptoms.

SW480 colon carcinoma cells express low levels of E-cadherin on the cell surfaces, corresponding with a high migratory activity (Masur *et al*, 2001). Although the DNA microarray data pointed towards a diminished E-cadherin expression at diabetogenic conditions, we were not able to confirm an additional reduction of the constitutively low E-cadherin cell surface expression (Figure 1A). For confirmation of the micro-array data, we also performed western blot analysis to detect changes of the total content of E-cadherin. Western blot analysis revealed a 30 and 25% reduced protein-expression levels of E-cadherin in both, MDA MB468 and SW480 cells, respectively (Figure 1B). Furthermore, we found increased numbers of VE- and N-cadherin transcriptoms, and also slightly increased VE- and N-cadherin cell surface levels, comparing physiological and diabetogenic culture conditions.

**Figure 2:**
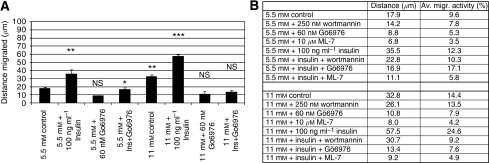
Analysis of the distance migrated of MDA MB468 breast cancer cells. Bars in (**A**) display the average distance (±s.e.m.) of 30 randomly selected cells of a 12 h observation period. Cells were cultured at either 5.5 mM or 11 mM glucose and treated with 100 ng ml^−1^ insulin, 250 nM wortmannin or a combination of both substances. (**B**) Depicts the distance migrated and the average migratory activity of MDA MB468 cells cultured at either 5.5 mM or 11 mM glucose with and without additional insulin (100 ng ml^−1^) and treated with the PI3K inhibitor wortmannin, the PKC*α* inhibitor Go6976, or the MLCK inhibitor ML-7. The results show mean values of three independent experiments. Statistical comparison between specified values was made by two-tailed unpaired *t*-test. ^*^P<0.05; ^**^P<0.01; ^***^P<0.001; NS, not significant.

In a first summary; altered levels of adhesion molecules did promote locomotion of the investigated cell lines. The important role of E-cadherin and PKC*α* in cell migration was already described by Masur *et al* (Masur *et al*, 2001). Therefore, we analysed signalling pathways of glucose and insulin leading to an activation of PKC*α* and finally to an increased cellular motility. Using the inhibitors wortmannin (PI3K inhibitor at 250 nM), Go6976 (PKC*α* inhibitor at 6 nM) and ML-7 (MLCK inhibitor at 10 *μ*M), we were able to track down the key molecules of the signalling cascade of the glucose- and insulin-induced tumour cell migration. Each of the three tested inhibitors showed a significantly reduced migratory activity of the glucose mediated-, the insulin-induced cell movement and also the combination of high glucose and high insulin (Figure 2A). Thereby, not only the percentage of moving cells was diminished by either wortmannin, Go6976 or ML-7, also the distance migrated and the duration of cellular movement were significantly reduced. Wortmannin, for example, only reduced the insulin-mediated increase of distance migrated down to control level of the matching glucose concentration, whereas no significant further reduction below the glucose-induced level could be observed (Figure 2B). However, both Go6976 and ML-7 diminished the insulin- and the glucose-mediated increase in tumour cell migration. As both PKC*α* and MLCK seemed to have central roles, we analysed the expression level of these key molecules by western blot analysis.

**Figure 3:**
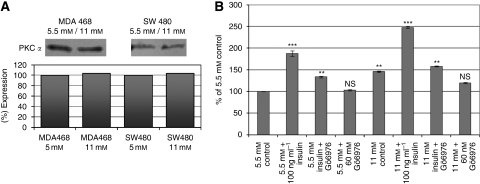
(**A**) Immunoblot analysis of PKC*α* expression of MDA MB468 breast cancer and SW480 colon carcinoma cell lines. Antibodies against the human PKC*α* isotype were used, and specifically bound antibodies were detected with the use of a secondary HRP-linked mouse anti-human antibody. The expression levels of all blots were evaluated using the Wasabi software version 1.4. (**B**) Analysis of the PKC kinase activity of SW480 colon carcinoma cell lysates. Tumour cells were cultured at 5.5 mM or 11 mM glucose alone or in combination with 100 ng ml^−1^ insulin. The PKC*α*-specific inhibitor Go6976 (6 nM) was added to control and insulin-treated samples in parallel. The phosphorylation of the PKC substrate (coated on 96-well plates) was detected by an HRP-conjugated secondary antibody, using a PKC kinase activity kit (StressXpress from Biomol). The graphs show mean values of three independent experiments (2.5 × E5 cells per sample). Statistical comparison between specified values was made either by two-tailed unpaired *t*-test or by ANOVA with Dunnett's multiple comparisons test. ^**^P<0.01; ^***^P<0.001; NS, not significant.

The expression levels of PKC*α* were not significantly increased when cells were cultured in hyperglycemic conditions (Figure 3A). Therefore, it was consequent to study the enzymatic activity of PKCs by measuring the phosphorylation of PKC substrates (StressXpress PKC kinase activity kit). For specificity control, we used the PKC*α* inhibitor Go6976. The PKC activity assay revealed a 23% increased activity of the total classical PKC enzymes in SW480 cells, comparing cells cultured at 11 mM glucose *vs* 5.5 mM (Figure 3B). The classical PKC activity could be inhibited by the PKC*α* inhibitor Go6976 at 6 nM, independent of the supplemented medium by glucose and insulin.

**Figure 4:**
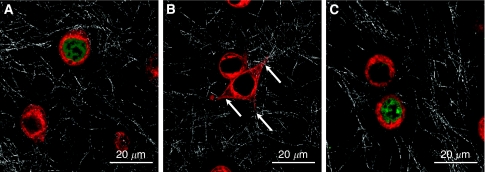
(**A**–**C**) PKC-specific dye rim-1 indicates that a translocation of PKCs at diabetogenic conditions can be reversed by Go6976. (**A**) Depicts untreated MDA MB468 cells embedded in a 3D collagen matrix. As indicated by rim-1 staining, the PKC is localised in the cytosol, whereas Sytox green was used for staining of the nucleii. Rim-1 staining of MDA MB468 cells stimulated with 11 mM glucose and 100 ng ml^−1^ insulin shows a translocation of PKC towards the membrane and the pseudopodia – indicated by the arrows in (**B**), whereas the PKC*α*-specific inhibitor Go6976 (6 nM) was able to diminish the insulin-mediated translocation (**C**).

To further illuminate the major role of the classical PKCs within the locomotory machinery, we stained the cells with the PKC-specific dye rim-1 (Figure 4A–C). Although in resting cells PKC staining/rim-1 is found in the cytosol (Figure 4A) and PKC activation by glucose and insulin led to a translocation of PKC to the membrane and into the pseudopodia (Figure 4B). Incubating MDA MB468 cells with the PKC*α*-specific inhibitor Go6976 prevented this translocation of PKC (Figure 4C).

**Figure 5:**
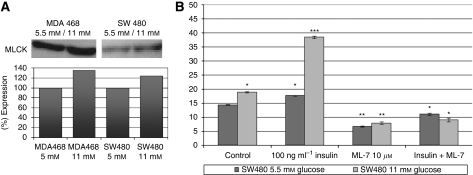
(**A**) Immunoblot analysis of MLCK expression of breast cancer (MDA MB468) and colon carcinoma (SW480) cell lines. Antibodies against the human myosin light chain kinase (MLCK) were used, and specifically bound antibodies were detected with the use of a secondary HRP-linked antibody. The expression levels of all blots were evaluated using the Wasabi software version 1.4. (**B**) Inhibition of the pro-migratory effects of glucose and insulin in SW480 cells by the MLCK-specific inhibitor ML-7. Migration of the cells was induced by incubating the cells with high glucose concentrations (11 mM) and by the addition of 100 ng ml^−1^ insulin. Locomotory activity and therefore the distance migrated was blocked by the addition of 10 *μ*M ML-7. The graphs show mean values of three independent experiments (90 cells were analysed per sample). ^*^P<0.05; ^**^P<0.01; ^***^P<0.001.

As the DNA microarray data showed significant elevated levels of non-muscle myosins and *MLCK* gene transcripts, we also analysed the MLCK protein expression level. Western blot analysis revealed a 30% increase of MLCK proteins in MDA MB468 cells at hyperglycemic conditions when compared with physiological conditions (Figure 5A). In SW480 cells, we found a more than 20 percent increase of MLCK expression. In addition, we performed migration studies testing the influence of the MLCK-specific inhibitor ML-7 (Figure 5B). High glucose (11 mM) as well as high glucose plus insulin (100 ng ml^−1^) led to an elevated migratory activity, resulting in an increased distance migrated. ML-7 at 10 *μ*M was able to reduce both, the glucose-mediated as well as the glucose- and insulin-mediated migratory activity of all tested cell lines.

## Discussion

Epidemiological studies revealed a correlation between metabolic alterations as found in people with obesity/adiposity and DM T2, and an increased risk of cardiovascular diseases (Eckel *et al*, 2002; Brown *et al*, 2008). These alterations found in obese people are best summarised by the metabolic syndrome, mainly characterized by high serum levels of glucose, insulin and triglycerides, as well as a chaotic pattern of cytokines. However, Frezza *et al* and Stattin *et al* showed details on the influence of obesity on the progression of cancer (Frezza *et al*, 2006; Stattin *et al*, 2007). However, there are data from 20 years ago, describing the positive effects of glucose and insulin on the progression of tumours (Stracke *et al*, 1988; Beckner *et al*, 1990), without further identifying the genetic and molecular modifications. Recently, we have compiled the molecular and epidemiological links between the metabolic syndrome and diabetes mellitus on the progression of cancer (Masur *et al*, 2008).

Established tumour cell lines are cultured routinely in media containing high amounts of glucose, for example, RPMI1640 11 mM, Mc Coy's 5A 22 mM and DMEM (high glucose) 25 mM. This plethora of glucose ensures the survival of most cells over long periods of cell culture without the need to monitor glucose concentration. However, these hyperglycemic conditions may lead to changes in cellular functions, such as carbohydrate and fatty acid metabolism, proliferation and cell motility.

Most of the aggressively growing tumours have common cellular characteristics, including high proliferation rates and the potential to invade peritumoural tissue. The increased proliferation and concomitantly high metabolic activity is often accompanied by an enhanced glycolysis and truncated mitochondrial respiration, also known as the Warburg Effect (anaerobic glycolysis) (Gatenby and Gillies, 2004). Those effects will be promoted by the above mentioned culture media.

Additionally, aggressively growing tumours are often characterized by an increased expression of GLUT-1 glucose transporters (Hauptmann *et al*, 2005), resulting in a high glucose uptake, a metabolic shift to anaerobe glycolysis and therefore an increased lactate production (Gillies and Gatenby, 2007; Kroemer and Pouyssegur, 2008). The tumour-associated Warburg effect has been linked to a poorer clinical outcome of tumour patients (Walenta and Mueller-Klieser, 2004; Kim and Dang, 2006). The increased anaerobic metabolism of glucose is followed by an elevated lactate secretion and acidification of the surrounding tumour tissue (Pelicano *et al*, 2006). The acidification causes an architectural deterioration of the ECM at the cutting edge of tumour growth and thereby creating space for an exponentially growing tumour mass. Furthermore, acidification may also lead to increased activation of MMP activities (Chaussain-Miller *et al*, 2006).

Obesity is often associated with increased serum glucose and triglycerides levels, as well as highly spiking insulin concentrations, which are risk factors for increasing the likelihood of tumour progression. Although the effects of obesity and high insulin as promoters of cancer cell proliferation are described for colon (Giovannucci, 2001), breast (Vona-Davis *et al*, 2007), endometrium and kidney (Hjartaker *et al*, 2008), little is known about the altered gene signatures with regard to cellular features like cell adhesion and migration. Therefore, we cultured tumour cell lines at physiological glucose concentrations (5.5 mM) and at high glucose (11 mM) with or without additional insulin (100 ng ml^−1^). Thereafter, we analysed gene activities of transcripts involved in cell cycle regulation, signal transduction, cell–cell contacts and motor proteins as parts of the locomotory machinery, comparing physiological and diabetogenic conditions.

At high insulin levels, we found higher number of transcripts of genes responsible for cell cycle processes, such as cyclin A1 and E regulation, the transition from G1 to S phase (see Table 1). Together with the elevated copy numbers of transcription factor E2F, these findings suggest increased proliferation rates as described for colon cancer after insulin stimulation (Giovannucci, 2001).

Apart from the mode of action of insulin on the tumour cells expressing insulin and IGF receptors, we found modulated gene activities for cell cycle regulation, cell-to-cell contact and locomotion when cells were cultured solely at high glucose (11 mM).

These genetic findings suggest a diversity of insulin and diabetogenic glucose concentrations modulating cell signalling processes involved in proliferation and cell migration. Okumura *et al* described increased PKC*α* levels when multi-drug resistant MCF-7 cells were cultured in high glucose media (Okumura *et al*, 2002). They detected that an elevation of PKC*α* protein content was followed by an increased content of DNA and elevated levels of cyclin D. Although cyclin D can be found in oscillating concentrations at almost all periods of the cell cycle, we found an insulin and glucose-dependent upregulation of cyclins A and E necessary for the transition from G1 to the S phase, which are more pronounced markers for proliferation.

Furthermore, analysing migratory behaviour of SW480 and MDA MB468 cells, we have shown that high levels of glucose-induced locomotory activity resulted in an extended time of locomotion (caused by a reduced number and duration of pauses), resulting in an elevated distance of migration. When cells were stimulated with insulin the pro-migratory effects were even stronger. These cell migration-promoting effects of glucose and insulin were the result of altered gene activities of several adhesion molecules (e.g. integrines and cadherins, see Figure 1), and an increased PKC*α* activity (Figure 3) resulting in an elevated activation of the locomotory machinery – namely MLCK.

The activity of the migratory machinery – over-fueled by high glucose (11 mM) and insulin concentrations (100 ng ml^−1^) – could be reversed when the media were supplemented by the pharmacological PKC*α*-specific inhibitor Go6976 (6 nM). These experimental data indicate that high glucose concentrations are able to induce PKCs – especially PKC*α* activity – functioning as a propulsive cue to induce tumour cell locomotion (Figures 3B and 4B).

Further clarifying the influence of PKCs at diabetogenic conditions, we used a StressXpress PKC Kinase Activity Assay Kit and found a strong activation of PKC enzymatic activities – especially PKC*α* – as determined by the PKC*α*-specific inhibitor Go6976 (3B). As both, the PKC enzymatic activity and the migratory activity of the cells, were inhibited by Go6976, this suggests that PKC*α* is activated at high glucose concentrations. Downstream of the increased PKC activities within the locomotory machinery, we also found elevated numbers of MLCK transcripts (tab 1) as well as increased MLCK protein levels (Figure 5A). The glucose- and insulin-promoted elevation of locomotion could be completely blocked by the MLCK-specific inhibitor ML-7 (Figure 5B).

We found a downregulation of E-cadherin and an upregulation of VE-cadherin at the transcriptional level as well as on the cell surface. Low E-cadherin expressing tumour cells are more proned to evade a tumour mass and to invade the surrounding tissue. The increased levels of VE-cadherin on tumour cells with low E-cadherin expression give the molecular advantages for extravasation. This tumour evasion process is further stressed by the upregulated collagen receptor (Table 1). Collagen is the main component of the ECM and collagen fibers are mandatory railroads for the locomotion of tumour and immune cells (Entschladen *et al*, 2005). Under hyperglycemic conditions, we observed not only an increased expression of the collagen receptor but also of the integrin-linked kinase and other kinases, regulating many cellular processes, including growth, proliferation, survival, differentiation, migration, invasion and angiogenesis (McDonald *et al*, 2008).

Hyperglycaemia and the Warburg effect lead to an increased glucose metabolism, an elevated lactate production and secretion, which cause the acidification of the tumour microenvironment. The acidification induces cell death of non-malignant tissues and thereby creating space for the growing tumour mass. A further advantage to facilitate cellular motility is the degradation of the ECM by the upregulation of metalloproteinase 7 and 10.

Our data strongly confirm a link between the epidemiological observations and the molecular modulators of cell proliferation and migration, how obesity and adiposity might influence the clinical course of a cancer patient who also deals with DMT2. In general, glucose has been seen as a food necessary for the supply of fast energy in order to satisfy the cells’ demand on ATP. In addition, our data indicate that glucose and insulin are directly involved in cell signalling processes, responsible for the regulation of proliferation and migration. Understanding the diverse roles of glucose and insulin – energy supply and modulators of signalling cascades – and delineating glucose and insulin signalling pathways should facilitate prevention and drug discovery research to control obesity, diabetes and cancer.

## Figures and Tables

**Table 1:** Glucose and insulin increase proliferation and migratory activity of different tumour cell lines

	**2.75 mM**	**5.5 mM**	**11 mM**	**22 mM**	**5.5 mM+insulin**	**11 mM+insulin**
*(A) Proliferation* (%) *in comparison to 5.5 mM glucose*						
HT29	89.8^*^	100.0	113.4^*^	125.5^**^	110.2^*^	126.7^**^
SW480	92.4^*^	100.0	134.6^**^	129.4^**^	105.8 NS	144.2^**^
MDA MB468	84.1^**^	100.0	117.8^*^	118.8^*^	108.9^*^	133.7^***^
MCF-7	92.5^*^	100.0	108.6 NS	115.1^*^	104.3 NS	123.3^**^
PC3	97.3 NS	100.0	104.3 NS	106.6^*^	102.7 NS	107.0 NS
T24	95.3 NS	100.0	106.8 NS	93.8 NS	109.0 NS	107.5^*^
						
*(b) Average migratory activity* (%)						
HT29	1.3	7.4	16.9	18.9	12.9	27.9
SW480	2.7	12.6	18.4	16.2	15.8	26.0
MDA MB468	6.8	9.6	14.4	17.4	12.3	24.6
MCF-7	5.5	18.8	23.5	19.2	24.0	29.4
PC3	12.2	12.0	17.3	16.3	17.9	37.5
T24	15.3	29.0	46.8	26.5	28.0	52.3
						
*(C) Distance migrated (μm)*						
HT29	7.8	12.4	15.6	18.4	17.7	16.0
SW480	3.5	10.5	16.1	19.8	17.8	29.2
MDA MB468	8.6	17.8	32.8	27.0	35.5	57.5
MCF-7	2.4	7.6	8.2	7.3	10.8	14.1
PC3	10.9	12.6	16.9	15.9	13.0	15.4
T24	18.8	26.1	48.7	20.7	25.8	51.8

Abbreviation: NS=not significant.

^*^P>0.05; ^**^P>0.01; ^***^P>0.001.

(A) End point determination of tumour cell proliferation after cultivation at either 5.5 mM glucose, 11 mM glucose or 11 mM glucose plus 100 ng ml^−1^ insulin. (1E5 cells per sample were seeded and cultured for 1 week). All samples are compared with the physiological glucose concentration of 5.5 mM (100%). (B) Comparison of the average migratory activity of tumour cell lines revealed increased activity with increased glucose levels (2.75 mM<5.5 mM<11 mM), but also differences between the cell lines with regard to the ability to further increase activity at very high glucose concentrations (22 mM). (C) 3D-migration assays revealed up to three-fold increase in distance migrated (e.g. MDA MB468) cells were treated with high glucose (5.5 mM *vs* 11 mM) and insulin (100 ng ml^−1^) concentrations. The table show mean values of three independent experiments (90 cells were analyzed per sample). Statistical comparison between specified values was made either by two-tailed unpaired *t*-test.

**Table 2:** Micro array analysis revealed alterations of gene activity by comparing physiological and diabetogenic conditions

	**5.5 mM vs 11 mM glucose**	**5.5 mM vs 11 mM glucose+insulin**
*Proliferation/cell cycle*		
Cyclin A1	14.09	11.13
Cyclin E2	4.90	2.39
E2F transcription factor 3	4.16	4.80
		
*Signal transduction*		
Integrin-linked kinase	3.92	4.85
Phosphoinositide-3-kinase	2.76	4.05
Protein kinase Cα	4.68	9.71
Protein kinase Cδ	4.55	5.01
MLCK	6.28	4.03
		
*Cell migration and adhesion*		
Integrin β1	5.10	3.71
Integrin α2	5.66	4.89
Myosin IA	4.27	6.37
Myosin IE	4.44	8.35
Myosin VIIA	4.31	11.90
Nonmuscle myosin II	3.75	4.07
N-cadherin (neuronal)	3.79	6.35
VE-cadherin (vascular)	2.90	5.76
Matrix metallopeptidase 7	3.41	3.55
Matrix metallopeptidase 10	2.94	2.26
TIMP metallopeptidase inhibitor 3	0.40	0.33
E-cadherin (epithelial)	0.22	0.25

Tumour cell mRNA was transcripted into cDNA and labeled with either Cy3 or Cy5. The activity of genes involved in cell migration/cell adhesion, in proliferation/cell cycle, and in signal transduction was investigated. The DNA microarray analysis was performed three times and only data with higher than 2.5-fold changes in gene activity were taken into consideration.
